# Polymer-Engineered MXene Composites for Durable Electrochemical Energy Storage: Suppressing Oxidation, Preserving Structure, and Extending Cycle Life

**DOI:** 10.3390/polym18111365

**Published:** 2026-05-31

**Authors:** Byeongji Beom, Man-Ki Moon, Jun-Hyeong Jung, Seung-Chan Jung, Eou-Sik Cho, Keun-A Chang, Jae-Hee Han

**Affiliations:** 1 Department of Materials Science and Engineering, Gachon University, Seongnam 13120, Republic of Korea; jjkk3239@gachon.ac.kr (B.B.); wangpeterpan@gachon.ac.kr (M.-K.M.); 701hoo@gachon.ac.kr (J.-H.J.); jsc4486@gachon.ac.kr (S.-C.J.); 2 Department of Electronic Engineering, Gachon University, Seongnam 13120, Republic of Korea; es.cho@gachon.ac.kr; 3 Department of Pharmacology, College of Medicine, Gachon University, Incheon 21999, Republic of Korea; keuna705@gachon.ac.kr

**Keywords:** MXene, polymer composites, electrochemical energy storage, oxidation stability, structural stability, cycle life, interfacial engineering, ion transport

## Abstract

Polymer-engineered MXene composites have emerged as a versatile materials platform for electrochemical energy storage, offering a means to address key limitations associated with ion transport, structural instability, and interfacial reactivity. This review provides a unified perspective on how polymer integration modifies the structure–transport–stability relationships of MXene-based systems across Na-ion batteries, aqueous Zn-ion batteries, and supercapacitors. In Na-ion systems, polymer-mediated interlayer engineering and porosity control improve ion accessibility and mitigate diffusion limitations arising from the large ionic radius of Na^+^. In aqueous Zn-ion systems, polymer electrolytes and interfacial layers regulate Zn^2+^ solvation and deposition behavior, suppressing dendritic growth and parasitic reactions. In supercapacitors, polymer–MXene hybrids establish coupled ionic–electronic transport pathways and mechanically compliant architectures, enabling stable electrochemical performance under high-rate and deformable conditions. Particular emphasis is placed on the underlying mechanisms responsible for suppressing oxidation, preserving structural integrity, and extending cycle life, including interfacial passivation, desolvation regulation, and structural confinement. These coupled effects govern long-term electrochemical stability across different energy storage systems. Finally, recent advances in operando characterization, data-driven materials design, and scalable processing are discussed in the context of future development. By linking material design strategies to fundamental mechanisms, this review outlines a coherent framework for the rational development of polymer–MXene composites toward practical energy storage applications.

## 1. Introduction

Electrochemical energy storage technologies underpin modern energy systems, supporting applications ranging from portable electronics and electric vehicles to large-scale grid stabilization. The growing demand for high energy density, fast charge–discharge capability, and long operational lifetime has driven intensive research into advanced electrode materials and interfacial engineering strategies. However, practical performance remains constrained by coupled physicochemical limitations, including sluggish ion transport, structural degradation during repeated cycling, and instability at electrode–electrolyte interfaces. These challenges are particularly severe in high-rate and long-term operation, where nonuniform ion flux, mechanical stress accumulation, and parasitic side reactions collectively accelerate performance decay and limit device reliability [1,2,3,4].

Two-dimensional (2D) materials have emerged as promising platforms for overcoming these limitations due to their high surface area and tunable physicochemical properties. Among them, MXenes, a family of transition metal carbides and nitrides derived from MAX phases, have attracted significant attention owing to their metallic conductivity, hydrophilic surfaces, and rich surface chemistry [5,6,7]. These features enable efficient electron transport and strong interfacial interactions with electrolyte species, facilitating rapid charge transfer and ion accessibility. Nevertheless, pristine MXene nanosheets suffer from intrinsic drawbacks, including restacking driven by van der Waals interactions and susceptibility to oxidation under ambient and electrochemical conditions, both of which reduce accessible active sites and degrade electrical conductivity over time [5,8,9]. These limitations underscore the need for integrated strategies that can simultaneously stabilize MXene structures while controlling interfacial processes.

In this context, the integration of MXenes with functional polymers has emerged as a highly effective approach for constructing robust and tunable electrochemical systems. Polymer–MXene composites combine the continuous electronic pathways provided by MXenes with the mechanical flexibility, chemical versatility, and ion-transport functionality of polymers [10,11]. Unlike conventional carbon-based conductive additives, MXenes possess abundant surface functional groups that enable strong hydrogen bonding and coordination interactions with polymer chains, resulting in improved dispersion, enhanced interfacial adhesion, and suppression of nanosheet aggregation [5,11]. More importantly, polymer matrices can actively regulate ion transport and interfacial chemistry by modulating local solvation environments, controlling ion flux distribution, and suppressing undesirable side reactions such as hydrogen evolution and interfacial corrosion [10,11,12,13].

The advantages of polymer–MXene systems become particularly evident in emerging battery chemistries where interfacial instability and ion transport limitations dominate performance. In sodium (Na)-ion batteries (SIBs), the larger ionic radius of Na^+^ leads to sluggish diffusion kinetics and pronounced volume changes, resulting in poor rate capability and structural degradation of electrode materials [14,15]. In aqueous zinc (Zn)-ion batteries (AZIBs), although Zn metal anodes offer high theoretical capacity and intrinsic safety, their practical implementation is hindered by dendrite formation, hydrogen evolution reactions, and interfacial corrosion, all of which compromise cycling stability and Coulombic efficiency [16,17,18,19]. In both systems, controlling ion desolvation, flux uniformity, and interfacial reactions is essential for achieving durable performance. Polymer–MXene composites offer an integrated materials platform for addressing these challenges by integrating structural confinement, interfacial regulation, and transport optimization within a single material framework. Through rational design of polymer chemistry and MXene surface terminations, it is possible to tailor ion transport pathways, stabilize interfacial structures, and mitigate degradation mechanisms across a wide range of electrochemical systems [10,11]. In addition, these hybrid materials enable the convergence of battery-type and capacitive energy storage behaviors, offering opportunities to bridge the gap between high-energy-density and high-power-density devices.

In this review, we present a comprehensive overview of recent advances in polymer–MXene composites for electrochemical energy storage. We first discuss the fundamental properties of MXenes and functional polymers, followed by their application in Na-ion batteries, aqueous Zn-ion batteries, and supercapacitors. Particular emphasis is placed on the mechanistic roles of interfacial chemistry, ion transport, and structural design. Finally, we outline key challenges and future research directions toward the development of high-performance, durable, and scalable polymer–MXene systems.

**Figure 1:**
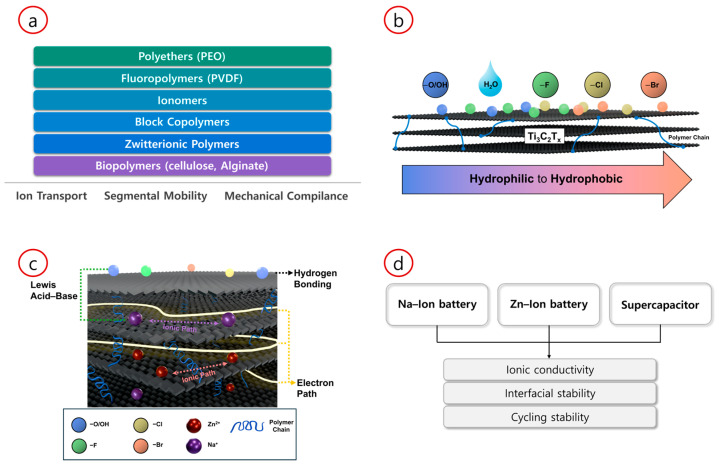
Design framework of polymer-engineered MXene composites for electrochemical energy storage. (**a**) Classification of functional polymers used in MXene-based composites, including polyethers, fluoropolymers, ionomers, block copolymers, zwitterionic polymers, and biopolymers, highlighting their roles in ion transport, segmental mobility, and mechanical compliance. (**b**) Schematic illustration of Ti_3_C_2_T_x_ MXene with representative surface terminations, showing how surface chemistry modulates interfacial polarity and governs the transition from hydrophilic to hydrophobic behavior. (**c**) Interfacial interactions and transport pathways in polymer–MXene composites, where ion-conducting polymer domains and electronically conductive MXene networks form coupled pathways (yellow dashed arrow), supported by hydrogen bonding (blue dashed arrow) and Lewis acid–base interactions (green dashed arrow). (**d**) Device-level implications of polymer–MXene design across Na-ion batteries, aqueous Zn-ion batteries, and supercapacitors, linking composite structure to enhanced ionic conductivity, interfacial stability, and cycling stability.

Figure 1 outlines the overall design framework of polymer–MXene composites, emphasizing how polymer selection, surface chemistry, and interfacial interactions collectively govern ion transport, structural stability, and device-level performance across batteries and supercapacitors. This framework serves as a conceptual basis for the structure–property relationships discussed in the following sections.

## 2. Fundamentals of MXenes and Functional Polymers

MXenes constitute a class of two-dimensional transition metal carbides, nitrides, and carbonitrides with a general formula of M_n+1_X_n_T_x_, where M denotes an early transition metal such as Ti, V, Nb, Mo, or W, and X represents carbon and/or nitrogen, and T_x_ corresponds to surface terminations such as –O, –OH, and –F [5,6,7]. The diversity of M-site chemistry enables MXenes to exhibit composition-dependent electronic structures, redox activities, ion adsorption behaviors, and electrochemical stabilities, indicating that polymer–MXene composites should not be considered only within the Ti_3_C_2_T_x_ framework. The electrochemical behavior of MXenes is strongly influenced by their surface termination groups, which affect wettability, ion adsorption, charge-transfer kinetics, and desolvation processes at the electrode/electrolyte interface [5,20,21,22]. In addition to surface functionalization, vacancy engineering has recently emerged as an effective strategy for improving ion transport and increasing the number of electrochemically active sites. Representative examples include ordered-vacancy W-based MXenes and vacancy-engineered V-based MXenes, both of which have shown enhanced electrochemical performance through facilitated ion diffusion and improved charge-storage characteristics [23,24,25,26]. In aqueous and hybrid electrolyte systems, these surface and interfacial characteristics strongly influence ion transport and electrochemical stability [5,9,12,13]. However, strong interlayer van der Waals interactions often induce restacking of MXene sheets, reducing ion accessibility and limiting electrolyte penetration. In addition, the susceptibility of MXenes to oxidation during storage or electrochemical operation remains a major challenge for maintaining long-term electrochemical performance [8,9].

Incorporating polymers provides an effective route to mitigate these intrinsic limitations. In polymer–MXene composites, polymers function simultaneously as interlayer spacers, ion-conducting media, and protective interphases [10,11]. By preventing restacking, polymer chains preserve ion-accessible galleries, while their segmental dynamics facilitate ion transport, particularly in solid-state and gel-based systems [10,11,27]. In addition, polymer coatings can stabilize MXene surfaces by suppressing oxidation and reducing exposure to reactive species [9,10,11]. The selection of polymer chemistry strongly governs composite behavior: conducting polymers such as polyaniline and polypyrrole contribute additional redox-active sites and enhance pseudocapacitive charge storage [4,28], whereas elastomeric and structural polymers impart mechanical flexibility and resilience required for deformable devices [11,28]. Bio-derived and functional polymers further introduce abundant coordination sites, enabling strong interfacial interactions with both MXenes and ionic species.

Polymer–MXene systems generally exhibit coupled electronic and ionic transport, where electrons are primarily conducted through interconnected MXene networks while ions migrate through polymer-rich regions. The resulting electrochemical behavior depends on factors such as MXene loading, nanosheet alignment, polymer mobility, and interfacial compatibility [10,11]. Interactions at the polymer–MXene interface, including hydrogen bonding, electrostatic attraction, and occasional covalent coupling, influence dispersion stability, mechanical integrity, and ion transport behavior. These interfacial interactions also contribute to suppressing parasitic side reactions and improving electrochemical stability across diverse electrolyte environments [9,10,11]. These interfacial and transport characteristics are closely related to the material design strategies adopted for different electrochemical applications, which are discussed in the following sections.

MXene/polymer composites can be fabricated through a range of approaches, including surface modification, polymer coating, ionogel and gel-polymer electrolyte formation, hydrogel-network construction, and polymer lamination. Different fabrication routes can substantially influence MXene dispersion, conductive-network formation, interfacial contact with polymer phases, and ion/electron transport behavior. Consequently, the preferred MXene/polymer composition often varies depending on the target electrochemical system [9,11,29,30].

In metal-ion battery systems, MXene/polymer compositions are generally designed to balance ionic conductivity, interfacial stability, and mechanical confinement. Wang et al. reported a Ti_3_C_2_T_x_ MXene-modified PVDF-HFP/PMMA gel polymer electrolyte for Na-ion batteries, where incorporation of 8 wt% Ti_3_C_2_T_x_ produced an ionic conductivity of 3.28 × 10^−3^ S cm^−1^, a Na^+^ transference number of 0.558, and an electrochemical stability window of 5.25 V. The corresponding Na_3_V_2_(PO_4_)_3_/GPE/Na cell retained 95% of its initial capacity after 300 cycles at 0.5 C, demonstrating improved ion transport and cycling stability through MXene incorporation [31].

For Zn-ion batteries, the polymer fraction plays an important role in mechanical confinement, ion-flux regulation, and dendrite suppression. Liu et al. reported MXene/cellulose nanofibril ionotronic dual-network hydrogel films for stable Zn anodes and showed that MXene/CNF hydrogel compositions can reduce Zn nucleation overpotential and improve long-term Zn plating/stripping stability [32]. In this system, the polymer-rich CNF network provides mechanical robustness and promotes more uniform ionic transport near the Zn surface, while MXene contributes electrical conductivity and interfacial functionality. These results highlight the need to consider conductivity, mechanical toughness, interfacial stability, and ion-flux homogeneity together when designing MXene/polymer systems for Zn-ion batteries [32].

The compositional requirements for supercapacitors differ from those of metal-ion batteries because high electronic conductivity, ion-accessible surface area, pseudocapacitive contribution, and mechanical flexibility must all be maintained within the electrode structure. Pan et al. reported an all-solid-state flexible supercapacitor based on an MXene-containing ionogel and a PANI electrode, where the ionogel formulation improved ionic conductivity, cycling stability, and operation across a wide temperature range [28]. In addition, polymer and gel components in MXene-based supercapacitors have frequently been used to reduce nanosheet restacking, improve flexibility, and facilitate ion diffusion, while interconnected MXene networks remain essential for electron transport and capacitive charge storage [33,34,35,36,37].

Several studies have further examined the influence of MXene/polymer ratio on supercapacitor performance. Mohammadi et al. prepared electrospun Ti_3_C_2_T_x_ MXene/PANI/PVDF freestanding electrodes with different MXene/PANI mass ratios and reported a specific capacitance of 895 F g^−1^ at 0.5 A g^−1^ for the composition containing 80/20 MXene/PANI [38]. Similarly, Nandy et al. investigated PEDOT:PSS/delaminated Ti_3_C_2_T_x_ composites with varying compositions and observed a maximum specific capacitance of 718.67 F g^−1^ at 3.5 A g^−1^ in 1 M H_2_SO_4_ for the sample containing 12 wt% MXene [39]. Overall, the preferred MXene/polymer ratio differs according to the target electrochemical system. Metal-ion batteries typically require compositions that support ion transport, interfacial stability, and mechanical confinement, whereas supercapacitors rely more heavily on conductive MXene frameworks together with polymer-derived pseudocapacitance, flexibility, processability, and ion accessibility [28,31,32,33,34,35,36,37,38,39].

## 3. Polymer-Engineered MXene Anodes for Na-Ion Batteries

Sodium (Na)-ion batteries (SIBs) have emerged as promising alternatives to lithium-ion systems due to the natural abundance and low cost of Na resources. However, their practical implementation remains hindered by fundamental challenges associated with the larger ionic radius of Na^+^, which leads to sluggish diffusion kinetics, pronounced volume changes during cycling, and structural instability of electrode materials [14,15,31]. In addition, the relatively weak interaction between Na^+^ ions and host lattices often results in poor intercalation reversibility and limited rate capability. These limitations necessitate electrode architectures that can simultaneously provide rapid ion transport, structural robustness, and stable interfacial environments.

**Figure 2:**
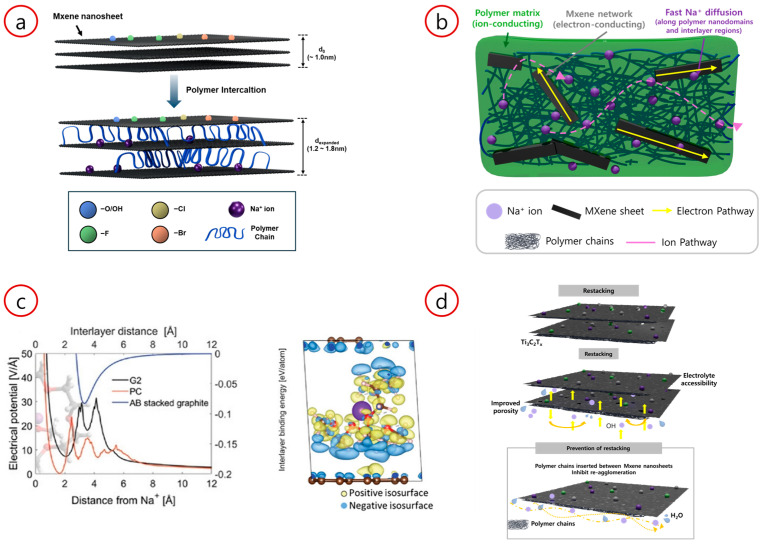
Structure–transport–stability relationships in polymer-engineered MXene anodes for Na-ion batteries. (**a**) Schematic illustration of polymer intercalation into Ti_3_C_2_T_x_ MXene galleries, showing expansion of the interlayer spacing and formation of ion-accessible channels for Na storage. (**b**) Na^+^ transport pathways in polymer–MXene composites, where ion-conducting polymer domains and electronically conductive MXene networks form coupled transport pathways that enable efficient ion diffusion and electron conduction. (**c**) Energetic aspects of Na^+^ insertion, illustrating the relationship between Na^+^–solvent interactions, interlayer binding, and ion accommodation within layered hosts. Adapted from Ferrero et al. [41], Chemical Reviews 2025, 125, 3401–3439 (CC BY 4.0). (**d**) Schematic representation of polymer-enabled suppression of MXene restacking, showing preservation of interlayer spacing (yellow arrows), improved porosity, and enhanced electrolyte accessibility during repeated cycling (orange arrows and purple balls).

MXenes, particularly Ti_3_C_2_T_x_, offer a compelling platform for SIB anodes owing to their layered structure, high electrical conductivity, and tunable surface chemistry [5,7]. These features enable efficient electron transport and ion accommodation; however, strong van der Waals interactions between nanosheets lead to severe restacking, reduced interlayer accessibility, and degraded electrochemical performance upon cycling [9,40]. Polymer engineering has therefore been widely introduced as a complementary strategy to regulate interlayer spacing, enhance interfacial compatibility, and construct continuous ion-transport pathways [10,11]. In polymer–MXene composites, polymers can function as intercalating spacers, flexible binders, or ion-conducting phases, thereby stabilizing the layered framework while preserving coupled ionic–electronic transport [10,11,27]. As illustrated in Figure 2, these design strategies establish intrinsic relationships between structure, ion transport, and electrochemical stability, which collectively determine the performance of MXene-based SIB anodes.

Figure 2 illustrates a comprehensive illustration of the structure–transport–stability relationships in polymer–MXene composites. As shown in Figure 2a, polymer intercalation increases the interlayer spacing of Ti_3_C_2_T_x_, generating additional ion-accessible galleries and alleviating intrinsic restacking. This structural modification enables more efficient Na^+^ accommodation within the layered framework. Figure 2b illustrates the formation of a dual-conduction network, in which polymer-rich domains facilitate Na^+^ transport while the MXene framework maintains continuous electron pathways. In addition to structural effects, Na storage is also governed by solvation and desolvation energetics, as highlighted in Figure 2c, where Na^+^–solvent interactions and interlayer binding determine the feasibility of ion insertion. Furthermore, polymer chains act as spacers that inhibit nanosheet re-agglomeration, preserving porosity and electrolyte accessibility, as depicted in Figure 2d. These combined effects contribute to improved ion transport kinetics and enhanced structural stability during repeated cycling.

### 3.1. Porosity Engineering and Interlayer Preservation

A primary limitation of MXene electrodes is the tendency of nanosheets to restack, which significantly reduces accessible surface area and restricts Na^+^ diffusion pathways. Porosity engineering is therefore widely employed to maintain open structures and enhance ion accessibility. By introducing polymeric spacers or constructing porous architectures, interlayer galleries can be preserved while facilitating electrolyte penetration into the electrode interior [10,11,40].

Within polymer–MXene composites, polymer chains inserted between adjacent nanosheets act as effective intercalating agents that prevent structural collapse during repeated sodiation and desodiation. This expanded interlayer configuration reduces diffusion resistance and improves rate capability, particularly under high current densities [10,11,31]. In parallel, porous MXene-based architecture, including aerogels and hydrogel-derived frameworks, provides interconnected ion transport channels while maintaining mechanical integrity [40,42,43]. Beyond simple spacing effects, pore distribution and connectivity critically influence electrochemical performance. Hierarchical porosity that integrates micro-, meso-, and macropores enhances electrolyte infiltration and shortens ion diffusion lengths, thereby improving both capacity and rate performance while mitigating stress accumulation associated with Na^+^ insertion [31,42,43].

Representative design strategies are summarized in Table 1, which provides a structured comparison of porosity engineering, interlayer expansion, and polymer-assisted transport regulation approaches. This summary highlights how different design motifs converge toward improving ion accessibility, structural integrity, and rate capability.

### 3.2. Polymer-Derived Carbon Coatings and Heterostructures

In addition to interlayer spacing control, polymer-derived carbon coatings provide an effective strategy to simultaneously enhance electrical conductivity and mechanical robustness in MXene-based anodes for Na-ion batteries. Polymers such as polyacrylonitrile (PAN), polydopamine (PDA), and polyethyleneimine (PEI) can be introduced as precursors and subsequently converted into conductive carbon layers through thermal treatment, forming conformal coatings on MXene nanosheets [10,11,44]. These carbon layers suppress nanosheet restacking, reinforce structural integrity, and establish continuous electron transport pathways across the electrode, thereby mitigating the intrinsic limitations of layered MXene frameworks during repeated sodiation and desodiation cycles.

Beyond simple coating, the formation of MXene–carbon heterostructures enables synergistic coupling between fast electronic conduction and preserved ion-accessible pathways. In such architectures, the carbon phase enhances electron mobility and structural resilience, while the MXene framework maintains interconnected ion transport channels, resulting in improved rate capability and cycling stability [40,44]. In addition, polymer-derived carbon layers can act as protective interphases that mitigate electrolyte decomposition and suppress interfacial degradation, thereby stabilizing electrode–electrolyte interactions under practical operating conditions. Recent studies further indicate that heteroatom-doped carbon coatings or chemically functionalized interphases can improve surface wettability and introduce additional electrochemically active sites, facilitating Na^+^ adsorption and diffusion. These modifications also contribute to more uniform charge distribution and reduced local stress accumulation, which are critical for maintaining long-term structural stability. These trends are consistent with the comparative framework outlined in Table 1, where polymer-derived carbon strategies are identified as key contributors to enhanced conductivity, structural integrity, and electrochemical durability in MXene-based SIB anodes.

### 3.3. Solvent Co-Intercalation and Segmental Dynamics

Beyond structural expansion and conductive heterostructure design, the regulation of Na^+^ transport is equally critical in determining the performance of MXene-based Na-ion anodes. In layered MXene systems, Na^+^ insertion is not governed solely by interlayer gallery size, but also by the energetics of ion solvation and desolvation, which strongly influence the feasibility and reversibility of intercalation. As highlighted in Figure 2c, Na^+^–solvent interactions and interlayer binding must be balanced to enable efficient insertion without destabilizing the layered host structure. In this context, polymer incorporation offers a direct means to tune the local solvation environment and facilitate ion migration within confined interlayer spaces [31,45,46]. Polymer electrolytes and gel-type polymer phases, particularly those containing polar functional groups and sufficient segmental mobility, promote salt dissociation, maintain intimate interfacial contact, and reduce charge-transfer resistance, thereby improving Na^+^ transport across the composite electrode [29,30,31,45]. At the same time, polymer-assisted ion-conduction pathways help distribute ion flux more uniformly, mitigating localized stress accumulation during repeated sodiation and desodiation. These transport-regulation effects are closely aligned with the comparative framework summarized in Table 1, where polymer-assisted solid/gel systems are distinguished not merely by mechanical support, but by their active role in stabilizing ion transport and preserving electrochemical reversibility. Collectively, these results demonstrate that high-performance polymer–MXene anodes for Na-ion batteries are achieved not through a single structural modification, but through coordinated control of interlayer accessibility, electron conduction, and polymer-regulated Na^+^ transport. For example, a Ti_3_C_2_T_x_ MXene-modified PVDF-HFP/PMMA gel polymer electrolyte containing 8 wt% MXene exhibited an ionic conductivity of 3.28 × 10^−3^ S cm^−1^, a Na^+^ transference number of 0.558, and an electrochemical stability window of 5.25 V. When applied in a Na_3_V_2_(PO_4_)_3_/GPE/Na cell, the system retained 95% of its capacity after 300 cycles at 0.5 C. These results show that MXene incorporation improves ion transport and cycling stability in polymer-electrolyte-based Na-ion batteries.

## 4. Interfacial Engineering for Durable Aqueous Zn-Ion Batteries

Aqueous Zn-ion batteries (AZIBs) have attracted considerable attention because of their low material cost, intrinsic safety, and the use of water-based electrolytes. Their practical deployment, however, remains limited by instability at the Zn metal anode, where dendritic growth, hydrogen evolution, corrosion, and nonuniform ion transport are tightly coupled failure processes [16,17,18,19,47]. These degradation pathways are closely linked to the local solvation environment of Zn^2+^, the desolvation barrier at the metal surface, and the mechanical stability of the electrode–electrolyte interface. In this context, polymer–MXene composites provide a particularly useful materials platform because they can regulate ion flux, reshape interfacial solvation, and introduce mechanically robust confinement within the same interphase [12,13,32].

**Figure 3:**
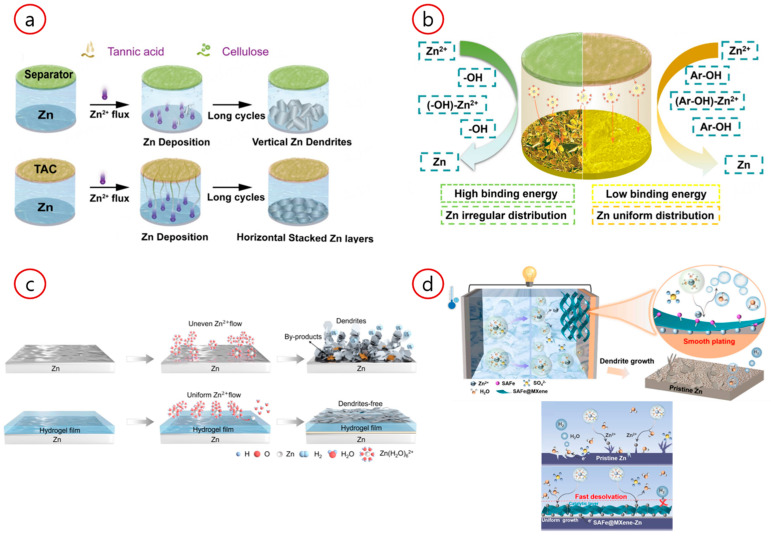
Interfacial stabilization strategies for polymer- and MXene-enabled Zn anodes in aqueous Zn-ion batteries. (**a**) Schematic illustration of a polymer electrolyte membrane at the Zn/electrolyte interface, suppressing direct contact between metallic Zn and the aqueous electrolyte and mitigating side reactions. (**b**) Regulation of Zn^2+^ solvation structure and interfacial coordination via polymer functional groups, enabling more uniform Zn nucleation and deposition. Both (**a**,**b**) are adapted from Deng et al. [12], *Energy Materials* 2025, 5, 500103 (CC BY 4.0). (**c**) Experimental comparison of Zn deposition behavior on bare Zn and MXene–cellulose nanofibril-protected Zn, demonstrating homogenized Zn^2+^ flux and effective dendrite suppression. Adapted from Liu et al. [32], *ACS Nano* 2025, 19, 13399–13413. (**d**) Schematic illustration of MXene-enabled interfacial catalytic regulation, highlighting accelerated Zn^2+^ desolvation, suppressed hydrogen evolution, and more uniform Zn deposition. Adapted from Zhang et al. [48], *Nano Letters* 2025, 25, 3756–3765 (Figures 1A and 4H,I) with permission.

Figure 3 summarizes the main stabilization pathways discussed in this section. Polymer electrolyte membranes and polymer-rich interphases can reduce direct contact between metallic Zn and bulk electrolyte, suppressing side reactions associated with water activity while guiding Zn^2+^ transport through coordinated functional groups. MXene-containing interphases extend this concept by adding electronically conductive and catalytically active domains that promote more uniform Zn deposition and faster interfacial kinetics. Taken together, these strategies show that durable Zn anodes are achieved not by suppressing a single degradation mode, but by coordinating interfacial chemistry, ion transport, and structural confinement during repeated plating and stripping.

As shown in Figure 3a,b, polymer electrolyte membranes regulate Zn^2+^ deposition by modifying the interfacial coordination environment and partially restructuring the solvation shell near the electrode surface [12,16]. Quantitatively, a 1% tannic-acid-modified cellulose polymer electrolyte membrane retained 83.1% of the discharge capacity after 1000 cycles at 5 C, suggesting that polymer-mediated Zn^2+^ redistribution contributes to improved long-term cycling stability. Functional groups such as hydroxyl, carboxyl, and ether moieties can coordinate with Zn^2+^, thereby lowering the kinetic barrier associated with desolvation and promoting uniform nucleation [12]. Figure 3c further provides experimental evidence that MXene–polymer hybrid interphases homogenize Zn^2+^ flux distribution and suppress localized current hotspots, effectively mitigating dendritic growth [32]. In a representative MXene–cellulose nanofibril hydrogel interphase, the MXene-CNF|Zn electrode showed a reduced nucleation overpotential of 19 mV, stable Zn∥Zn cycling for over 2700 h, and a high capacity of 323 mAh g^−1^ in Zn∥MnO_2_ cells. These results suggest that coupled mechanical confinement and more uniform ion/electron-field distribution contribute to improved Zn anode reversibility. Beyond these effects, MXene-containing interphases introduce additional catalytic and electronic contributions. As illustrated in Figure 3d, MXene-based layers can accelerate Zn^2+^ desolvation, suppress hydrogen evolution, and facilitate uniform Zn deposition through coupled ion-transport and interfacial reaction regulation [48]. Collectively, these observations indicate that stable Zn anodes are achieved through coordinated control of solvation structure, ion flux, and interfacial stability rather than by addressing individual degradation modes in isolation [16,17,18,19].

### 4.1. Suppression of Hydrogen Evolution and Dendrite Growth

Suppressing hydrogen evolution reaction (HER) and dendrite formation is central to improving Zn anode reversibility in aqueous systems. Polymer membranes and gel-type interphases can lower the activity of free water near the Zn surface, reduce parasitic water reduction, and regulate the local coordination environment of Zn^2+^ [12,13,16]. Functional groups such as hydroxyl, carboxyl, and ether moieties act as coordination sites that moderate desolvation and promote more uniform Zn nucleation, while the polymer framework redistributes local current density and resists protrusion growth. When MXene nanosheets are incorporated into these interphases, the resulting hybrid network further improves ion-flux homogeneity and mechanical stability. In particular, MXene–cellulose nanofibril composite hydrogels have shown that simultaneous ion-conduction guidance and structural confinement can effectively suppress dendritic deposition and stabilize Zn plating/stripping over prolonged cycling [32]. These results support the view that HER suppression and dendrite control should be treated as coupled interfacial problems rather than as separate design targets. Representative interfacial engineering strategies and their corresponding mechanistic roles in stabilizing Zn anodes are summarized in Table 2.

### 4.2. Advanced Polymer-Inorganic Hybrid Interphases

A second design route relies on hybrid interphases that combine polymer phases with inorganic or MXene-containing components. In these architectures, the rigid inorganic phase contributes interfacial stability and resistance to localized deformation, while the polymer phase maintains conformal contact and accommodates repeated volume changes at the Zn surface [10,13]. Such rigid–soft interphases act as mechanically stable barriers that redistribute ion flux, suppress corrosion, and reduce the likelihood of dendrite initiation. The same principle applies to MXene-enabled hybrid layers, where conductive nanosheets provide continuous electronic pathways and chemically active surfaces, while surrounding polymer domains regulate local solvation and ion coordination. This complementary design is effective because it couples mechanical integrity with electrochemical regulation rather than treating them as independent material functions.

### 4.3. Mechanistic Insights into Zn Solvation and Transport

The behavior of Zn anodes in aqueous electrolytes is fundamentally governed by the solvation structure of Zn^2+^ and its transformation during interfacial charge transfer. Because Zn^2+^ is strongly hydrated in bulk solution, desolvation at the electrode surface introduces a substantial kinetic penalty and simultaneously influences side reactions such as hydrogen evolution [16,18,19,50]. Polymer-containing interphases can tune this process by constraining solvent mobility, lowering local water activity, and providing coordination sites that guide Zn^2+^ migration toward more uniform deposition. MXene-containing interphases further extend this effect by contributing electronically conductive and catalytically active sites that accelerate desolvation and stabilize Zn deposition behavior [48]. Crucially, Zn anode stabilization cannot be reduced to a single parameter such as coating thickness or mechanical strength. Rather, long-lived AZIB performance requires simultaneous control of solvation chemistry, ion-flux distribution, interfacial reactivity, and mechanical confinement. Polymer–MXene composites are effective precisely because they integrate these functions within one interfacial design framework.

## 5. High-Performance Supercapacitors Based on Polymer–MXene Composites

Supercapacitors are attractive energy-storage devices because of their high-power density and long cycle life. However, their relatively low energy density compared with batteries necessitates electrode architectures that can sustain rapid ion transport while maintaining structural integrity during repeated cycling [1,2,4,33]. MXenes have emerged as highly promising electrode materials owing to their metallic conductivity, hydrophilic surfaces, and abundant redox-active transition metal sites, which collectively enable charge storage through a combination of electric double-layer capacitance and surface-controlled pseudocapacitive processes [1,4,34,35], enabling rapid surface redox reactions with minimal diffusion limitation. Nevertheless, pristine MXene films are prone to severe restacking driven by van der Waals interactions, resulting in reduced ion-accessible surface area, sluggish ion diffusion, and compromised mechanical compliance [36,37]. These intrinsic limitations become more pronounced in flexible and high-loading configurations, where interlayer collapse and transport bottlenecks directly degrade electrochemical performance.

**Figure 4:**
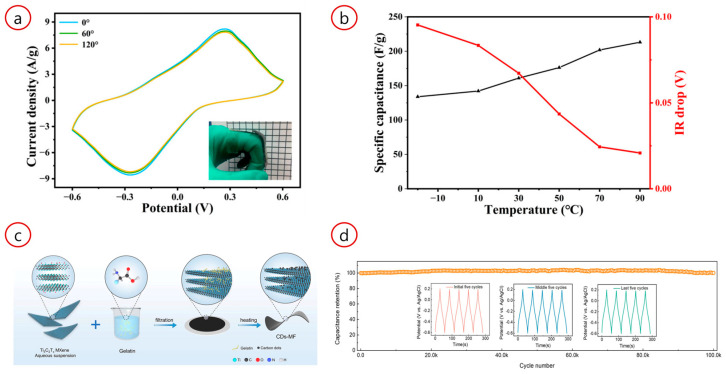
Representative polymer–MXene supercapacitor architectures and their electrochemical characteristics. (**a**) Bending performance of a flexible MXene-based all-solid-state supercapacitor, demonstrating retention of electrochemical response under mechanical deformation. (**b**) Temperature-dependent electrochemical behavior of the same device, showing stable operation across a wide temperature range enabled by an ionogel electrolyte. Black line is specific capacitance, and red line is internal resistance (IR) drop. Both (**a**,**b**) are adapted from Pan et al. [28], Molecules 2023, 28, 1554 (CC BY 4.0). (**c**) Schematic fabrication of a carbon-dots-intercalated MXene (CDs-MF) film electrode, illustrating spacer-assisted interlayer engineering. (**d**) Long-term cycling stability of the intercalated MXene electrode, demonstrating improved long-term cycling stability (Tested in 10 A/g, CDs-MF-2). Both (**c**,**d**) are adapted from Yang et al. [51], Energy Materials 2025, 5, 500006 (CC BY 4.0).

Integrating polymers into MXene electrodes provides an effective route to overcome these constraints by simultaneously regulating interlayer structure, interfacial chemistry, and ion transport pathways. As illustrated in Figure 4, polymer–MXene supercapacitor architectures establish coupled relationships between structural stabilization, ion accessibility, and electrochemical durability. In particular, flexible all-solid-state configurations based on ionogel electrolytes maintain stable electrochemical responses under mechanical deformation and across a wide temperature range, as evidenced in Figure 4a,b [28]. These systems benefit from the intrinsic flexibility of polymer networks and their ability to sustain ionic conductivity under varying environmental conditions. In parallel, interlayer engineering strategies that incorporate nanoscale spacers or carbonaceous domains effectively suppress restacking and preserve ion transport channels, as demonstrated in Figure 4c,d [51]. Collectively, these examples show that polymers function not merely as passive binders but as active structural and transport regulators that govern the electrochemical behavior of MXene-based supercapacitors.

### 5.1. Synergistic Conductive Polymer Hybrids

The integration of MXenes with conductive polymers such as PEDOT:PSS, polyaniline, and polypyrrole establishes a dual-conduction framework in which the polymer phase contributes redox-active sites and mechanical flexibility, while the MXene network maintains continuous electron transport pathways [1,2,4]. This synergistic interaction enhances charge storage through combined faradaic and capacitive mechanisms while reducing the need for additional conductive additives. Strong interfacial interactions, including hydrogen bonding between MXene surface terminations (–O, –OH, –F) and polymer chains, promote homogeneous dispersion and suppress nanosheet aggregation, resulting in improved structural integrity and stable electrochemical performance [4,11].

Beyond simple blending, structural engineering through spacer incorporation has emerged as a critical design strategy. The introduction of nanoscale spacers, including carbon dots and other polymer-derived nanoscale domains, expands interlayer spacing, suppresses restacking, and generates percolating ion-transport pathways throughout the electrode [1,51]. These modifications increase the density of electrochemically accessible sites while preserving electronic conductivity, enabling high-rate capability and long-term cycling stability. Consequently, polymer–MXene hybrid electrodes operate within a pseudocapacitive regime in which fast surface redox reactions and intercalation-driven charge storage coexist, thereby providing a balanced route toward high-rate and durable supercapacitor electrodes.

### 5.2. Flexible and Stretchable Architectures for Wearable Devices

The rapid development of wearable and deformable electronics has driven the demand for flexible supercapacitors capable of maintaining electrochemical performance under mechanical deformation. MXene-based films and composite papers provide a suitable platform owing to their intrinsic electrical conductivity and layered structure, while polymer components introduce mechanical compliance that accommodates bending, folding, and stretching [37,52]. In such architectures, maintaining structural integrity and interfacial adhesion between conductive networks and polymer matrices is essential for preserving volumetric capacitance under strain. The composition-dependent trade-off is evident in Ti_3_C_2_T_x_/PVA films: increasing the PVA fraction decreased the electronic conductivity from 240,238 ± 3500 S m^−1^ for pristine Ti_3_C_2_T_x_ to 22,433 ± 1400 S m^−1^ at 90 wt% Ti_3_C_2_T_x_/PVA and 0.04 ± 0.003 S m^−1^ at 40 wt% Ti_3_C_2_T_x_/PVA, while the tensile strength increased up to 91 ± 10 MPa. These results reflect the trade-off between conductivity, mechanical compliance, and ion accessibility in Ti_3_C_2_T_x_/PVA systems.

Flexible MXene-based supercapacitors demonstrate stable electrochemical responses under repeated mechanical deformation, as shown in Figure 4a, where capacitance retention is maintained during bending cycles [28]. In addition, ionogel-based electrolytes enable stable operation across a wide temperature range by maintaining ionic conductivity under both ambient and sub-zero conditions, as illustrated in Figure 4b [28]. For the MXene/MSA ionogel–PANI system, the PAIM-4 ionogel reached an ionic conductivity of 36.4 mS cm^−1^ at 90 °C, while the assembled all-solid-state supercapacitor delivered a mass-specific capacitance of 204.6 F g^−1^ and retained 91.56% of its capacitance after 10,000 cycles over a wide operating window from −20 °C to 90 °C. These results highlight the importance of maintaining mechanical stability, continuous ion-transport pathways, and interfacial robustness in flexible energy-storage systems. Polymer engineering can further help suppress crack propagation and preserve conductive pathways under repeated mechanical deformation.

### 5.3. Three-Dimensional Assembly and Interlayer Engineering

For MXene-based supercapacitors to achieve high-rate performance at practical electrode thicknesses, precise control over three-dimensional architecture and nanoscale transport pathways is essential. A major limitation of dense MXene films is restacking, which reduces electrolyte accessibility and slows ion diffusion through the layered structure [36,37]. Three-dimensional porous assemblies provide an effective solution by introducing interconnected ion-transport channels while preserving electronic conductivity across the MXene framework. In the carbon-dots-intercalated MXene film (CDs-MF) system, gelatin was introduced into MXene galleries at 3, 5, and 10 wt% and subsequently carbonized to form interlayer carbon dots. The optimized CDs-MF-2 electrode maintained a high electrical conductivity of 3493 ± 454 S cm^−1^ while delivering 396.4 F g^−1^ at 1 A g^−1^ and 1153.2 F cm^−3^, with no capacitance decay over 100,000 cycles [51]. These observations suggest that moderate interlayer disorder can improve ion accessibility while preserving electronic transport pathways.

Hierarchically porous MXene structures, including aerogels and hydrogel-derived networks, enhance electrochemical utilization by shortening ion diffusion distances and increasing the fraction of accessible surface sites [36,53]. These architectures function not simply as high-surface-area electrodes but as transport-regulated frameworks in which pore topology, interlayer connectivity, and mechanical stability collectively determine capacitive performance. At the nanoscale, interlayer engineering remains equally important; as shown in Figure 4c, the incorporation of molecular spacers expands interlayer galleries and reduces structural restacking while maintaining efficient electron transport through the MXene network [51]. The electrochemical consequence of this structural control is evident in Figure 4d, where intercalated MXene electrodes exhibit sustained cycling stability over extended operation [51]. From a mechanistic perspective, high-rate pseudocapacitive behavior in MXenes depends on rapid ion access to redox-active surfaces combined with continuous electronic conduction pathways. Accordingly, rational electrode design must balance porosity, interlayer accessibility, and structural retention during repeated cycling, emphasizing that high-performance MXene supercapacitors require integrated control of multiscale structure and transport properties rather than isolated optimization of individual parameters.

## 6. Mechanisms of Oxidation Suppression and Structural Preservation

**Figure 5:**
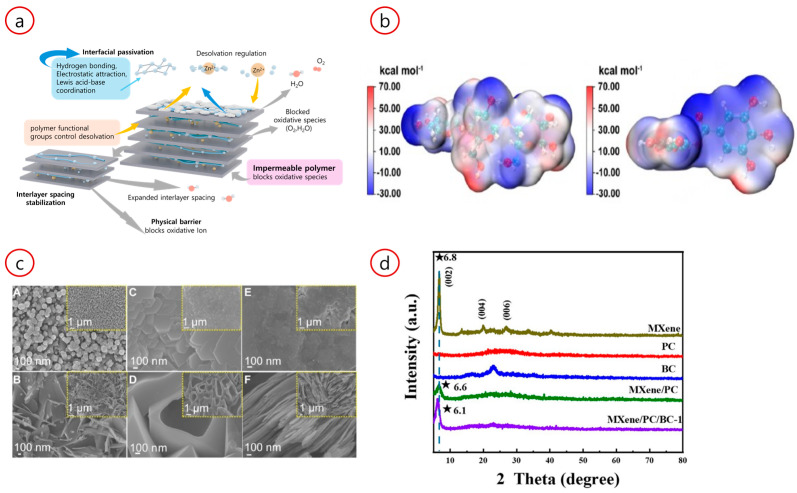
Mechanistic framework of polymer-enabled stabilization in MXene-based electrochemical systems. (**a**) Schematic illustrating the coupled roles of polymer incorporation in MXene architectures, including interfacial passivation via hydrogen bonding, electrostatic interactions, and Lewis acid–base coordination, regulation of Zn^2+^ desolvation, suppression of oxidative reactions involving O_2_ and H_2_O, expansion of interlayer spacing, and physical barrier effects that collectively stabilize electrochemical interfaces. The colored arrows and lines indicate the specific stabilization pathways enabled by polymer incorporation: yellow arrows represent polymer-functional-group-mediated Zn^2+^ desolvation/interfacial regulation, blue arrows and lines denote interfacial passivation and polymer-MXene interactions through hydrogen bonding, electrostatic interactions, and Lewis acid-base coordination, and gray arrows indicate the blocked transport or reaction pathways of oxidative species such as O_2_ and H_2_O. (**b**) Electrostatic potential distribution and coordination environment of Zn^2+^ species interacting with polymer-functionalized domains, highlighting modulation of binding strength and ion–solvent interactions. Adapted from Deng et al. [12] with permission. (**c**) Scanning electron microscopy (SEM) images (**A**–**F**) comparing Zn deposition morphologies under different interfacial conditions, demonstrating suppression of dendritic growth and formation of more uniform Zn layers. It shows the change in the Zn anode after prolonged charging and discharging. Adapted from Deng et al. [12] with permission. (**d**) X-ray diffraction (XRD) patterns showing a lower-angle shift in the MXene (002) reflection upon incorporation of polymer-derived spacers. The shift corresponds to increased interlayer spacing, indicating improved ion accessibility within the MXene-based architecture. Adapted from Liu et al. [54] (CC BY 4.0).

The performance improvements discussed in Section 3, Section 4 and Section 5 originate from the ability of polymer–MXene architectures to stabilize chemically reactive MXene surfaces while preserving ion-accessible structures under electrochemical operation. MXenes are inherently susceptible to oxidation due to their chemically active surface terminations and the presence of undercoordinated transition metal sites at edges and defects. Exposure to oxygen and moisture leads to the formation of insulating oxide species (e.g., TiO_2_), which degrade electrical conductivity and disrupt ion transport pathways [8,9]. Polymer incorporation fundamentally alters this behavior by introducing coupled stabilization mechanisms that operate across molecular, interfacial, and structural scales, enabling sustained electrochemical performance across Na-ion, Zn-ion, and supercapacitor systems. These coupled mechanisms are summarized in Figure 5.

To provide an overview of the relationships among the detailed mechanisms, Table 3 summarizes the stabilization functions of polymer components in MXene-based Na-ion and Zn-ion battery systems. Instead of presenting each function as a separate and unrelated effect, the table organizes polymer-mediated stabilization into mechanisms that are commonly shared across systems and those that are specific to each battery chemistry. In both Na-ion and Zn-ion systems, polymer incorporation contributes to interfacial passivation, chemical stabilization of reactive MXene surface terminations, suppression of oxygen and water diffusion, preservation of structural integrity, and regulation of coupled ion/electron transport pathways. These shared effects collectively serve as a general foundation for oxidation suppression and long-term electrochemical stability. By contrast, the dominant system-specific roles are determined by the working ion chemistry and the main degradation processes. In Na-ion batteries, polymer components mainly help maintain interlayer galleries and promote Na^+^ transport through expanded ion-accessible channels. In Zn-ion batteries, they regulate Zn^2+^ solvation/desolvation behavior, homogenize ion flux, and suppress dendritic deposition.

### 6.1. Interfacial Passivation and Chemical Stabilization

At the molecular level, polymer incorporation modifies the interfacial chemical environment of MXene surfaces, reducing their reactivity toward oxygen and water. Strong interfacial interactions, including hydrogen bonding, electrostatic attraction, and Lewis acid–base coordination, anchor polymer chains to surface terminations (–O, –OH, –F), effectively passivating reactive sites and limiting direct exposure of transition metal atoms to oxidative species [9,21,55]. This interfacial passivation suppresses the nucleation and growth of oxide domains, thereby preserving electrical conductivity and maintaining electrochemical activity. The effectiveness of polymer layers in suppressing MXene oxidation-related degradation has been supported by quantitative aging studies [57]. Kumar et al. investigated Ti_3_C_2_T_x_ MXene films passivated with an approximately 50 nm-thick UV-curable NOA65 polymer layer. During 180 days of ambient aging, the relative sheet resistance of the non-passivated MXene electrode increased by approximately 800%, whereas that of the polymer-passivated MXene increased by only approximately 20%. In addition, the transmittance of the non-passivated MXene film increased from approximately 85% to 89% after 180 days, which was attributed to the conversion of oxidized Ti_3_C_2_T_x_ into TiO_2_, while the polymer-passivated film exhibited almost negligible transmittance change. These results indicate that polymer passivation can substantially suppress oxidation-induced electrical and optical degradation of Ti_3_C_2_T_x_ MXene.

Lee et al. further demonstrated the protective effect of polymer lamination using poly(4-vinylphenol) (PVPh)-laminated Ti_3_C_2_T_x_ MXene electrodes [58]. The PL-MXene electrode consisted of an approximately 17.8 nm-thick MXene layer covered by an approximately 62 nm-thick PVPh layer, while maintaining an optical transmittance of approximately 76% at 550 nm. Under ambient air exposure, the relative resistance change in bare MXene increased to approximately 310% after 200 h and 470% after 600 h. In contrast, PVPh-laminated MXene electrodes showed much smaller resistance changes of 27–38% after 200 h and only slight additional increases after 600 h. XPS analysis also revealed that the TiO_2_ component in bare MXene increased approximately 95-fold after 7 days of air exposure, whereas the PL-MXene electrode showed little trace of TiO_2_ peaks and negligible changes after exposure. Under accelerated aging conditions of 70 °C and 50% RH, bare MXene exhibited an approximately 600% increase in ΔR/R_0_ after 200 h, whereas PL-MXene electrodes showed only 35–60% changes. These findings show that polymer overlayers can effectively suppress oxygen- and moisture-induced degradation while preserving the electrical and structural stability of MXene electrodes.

In parallel, polymer functional groups regulate ion solvation and interfacial coordination. In aqueous systems, functional groups such as hydroxyl, carboxyl, and ether moieties interact with Zn^2+^ ions, modifying the local solvation structure and lowering the kinetic barrier associated with desolvation. This effect promotes more uniform nucleation and deposition behavior while suppressing parasitic side reactions [12,55]. The modulation of ion–solvent interactions and coordination environments corresponds to the desolvation-regulation mechanisms illustrated in Figure 5a,b, where polymer chemistry governs both binding strength and interfacial reaction pathways.

### 6.2. Physical Barrier Effects and Morphological Stabilization

Beyond chemical stabilization, polymer matrices provide a physical barrier that suppresses the diffusion of oxygen, water, and other reactive species toward MXene surfaces. When MXene nanosheets are embedded within polymer frameworks, the transport of oxidizing species is kinetically hindered, particularly in dense coatings, crosslinked networks, and gel electrolyte systems [17,18,55]. This barrier effect becomes increasingly important under practical operating conditions, where electrodes are continuously exposed to ambient environments or aqueous electrolytes.

Polymer incorporation also plays a critical role in stabilizing deposition morphology and suppressing structural degradation. In pristine systems, localized ion flux and uneven interfacial reactions often lead to dendritic growth and surface roughening. In contrast, polymer–MXene hybrid interfaces regulate ion distribution and provide mechanical confinement, resulting in more uniform deposition behavior. As evidenced by the representative morphologies in Figure 5c, such interfacial regulation effectively suppresses dendrite formation and promotes stable plating/stripping processes. These combined effects demonstrate that physical barrier functions and ion-flux homogenization are essential for maintaining long-term interfacial stability.

### 6.3. Structural Confinement and Coupled Transport–Stability Relationships

Structural preservation in MXene-based systems is further governed by interlayer engineering and transport regulation. In pristine MXene films, strong interlayer interactions drive irreversible restacking, which reduces accessible surface area and restricts ion diffusion pathways. Polymer intercalation introduces steric hindrance and mechanical spacing between nanosheets, preserving interlayer galleries and maintaining ion-accessible channels during repeated cycling. The structural expansion enabled by polymer incorporation is directly linked to improved ion transport behavior. As shown in Figure 5d, the introduction of polymer-derived spacers increases interlayer spacing and enhances ion accessibility, thereby reducing diffusion resistance and improving electrochemical utilization. At the nanoscale, this interlayer regulation supports rapid ion transport while maintaining continuous electron conduction through the MXene framework.

At a fundamental level, stabilization of MXene-based electrodes reflects a coupled transport–stability relationship. Ionic conduction is preferentially facilitated within polymer-rich domains, while electronic transport is maintained through the MXene network, reducing localized current densities and mitigating electrochemical hotspots that accelerate degradation. This spatial and functional decoupling of ion and electron transport pathways enables stable operation across diverse electrochemical systems, including Na-ion batteries, Zn-ion batteries, and supercapacitors [1,4,31,55,56]. Taken together, these observations indicate that polymer engineering transforms MXenes from chemically reactive layered materials into stabilized electrochemical frameworks in which interfacial chemistry, ion transport, and structural integrity are tightly integrated. The mechanistic framework presented in Figure 5 provides a unified interpretation of these effects and establishes a direct linkage between interfacial chemistry, ion transport regulation, and long-term structural durability.

## 7. Future Perspectives and Advanced Characterization

The mechanistic framework established in Section 6 highlights that the stability of polymer–MXene systems is governed by tightly coupled interactions between interfacial chemistry, ion transport, and structural confinement. Future progress will therefore rely on directly probing these coupled processes under realistic operating conditions and translating mechanistic insights into predictive design strategies. In this context, advanced characterization, data-driven modeling, and scalable synthesis will play complementary roles in accelerating the development of robust MXene-based energy storage systems.

### 7.1. Operando Characterization of Dynamic Interfaces

A detailed understanding of dynamic interfacial processes during electrochemical operation is essential for identifying degradation pathways and validating the stabilization mechanisms discussed in Section 6. Conventional ex situ techniques provide only static snapshots, whereas operando approaches enable real-time observation of structural and chemical evolution under working conditions [59]. Techniques such as operando Raman spectroscopy, X-ray absorption spectroscopy, and in situ electron microscopy have revealed key processes, including ion intercalation, interfacial reconstruction, and phase transformations in MXene-based electrodes [20,59].

In polymer–MXene systems, operando analysis is particularly important for resolving how interfacial passivation, desolvation regulation, and structural confinement evolve during repeated cycling. Direct observation of changes in interlayer spacing, polymer segmental dynamics, and ion coordination environments can provide critical validation of the coupled transport–stability relationships proposed in Figure 5. Such insights are critical for bridging the gap between mechanistic understanding and rational material design.

### 7.2. Data-Driven Materials Design

The design space of polymer–MXene composites is inherently high-dimensional, encompassing polymer chemistry, MXene surface terminations, interlayer structure, and processing conditions. This complexity limits the effectiveness of conventional trial-and-error optimization approaches. Data-driven methodologies offer a practical route to accelerate materials discovery and design.

Machine learning frameworks capable of capturing nonlinear relationships between molecular structure and macroscopic properties have been increasingly explored in materials science [60]. These approaches have the potential to guide the selection of polymer compositions, crosslinking densities, and MXene loading levels for targeted performance metrics such as ionic conductivity, mechanical stability, and cycle life [61]. Importantly, the integration of experimental datasets with computational modeling enables closed-loop optimization, in which predictions are iteratively refined through validation. Such approaches are particularly relevant for polymer–MXene systems, where subtle variations in interfacial chemistry can lead to significant changes in transport behavior and stability. The development of reliable, high-quality datasets therefore remains a key prerequisite for realizing the full potential of data-driven design.

### 7.3. Scalable Manufacturing and Sustainability

For practical implementation, polymer–MXene composites must be produced using scalable and environmentally responsible methods. The replacement of hazardous HF-based etching processes with safer alternatives, including molten-salt or hydrothermal synthesis routes, represents a critical step toward industrial viability [11]. At the same time, the design of polymer matrices must consider not only electrochemical performance but also processability, cost-effectiveness, and recyclability.

Sustainability considerations are increasingly shaping the development of energy storage materials. Strategies aligned with material circularity, such as recycling, recovery, and reuse, are being explored to minimize environmental impact and extend material lifetimes [62]. In particular, the recovery of MXene nanosheets from end-of-life devices and their reintegration into secondary applications represents a promising but still emerging pathway toward sustainable energy storage systems. However, challenges remain in maintaining material quality during recycling and ensuring compatibility between recovered MXenes and newly synthesized polymer matrices. Addressing these issues will require coordinated advances in materials design, process engineering, and lifecycle assessment.

## 8. Conclusions and Outlook

Polymer-engineered MXene composites represent a versatile platform for advanced electrochemical energy storage. By integrating highly conductive MXene networks with polymer-mediated control of interfacial chemistry and structure, these systems address key limitations associated with ion transport, structural instability, and surface reactivity across Na-ion batteries, aqueous Zn-ion batteries, and supercapacitors.

In Na-ion systems, polymer-enabled interlayer engineering and porosity control mitigate sluggish ion diffusion and accommodate volume changes, enabling improved rate capability and cycling stability. In aqueous Zn-ion systems, polymer electrolytes and interfacial layers regulate Zn^2+^ solvation and deposition behavior, suppressing dendrite formation and parasitic reactions. In supercapacitors, polymer–MXene hybrids establish coupled ion–electron transport pathways and mechanically compliant architectures that sustain high-rate pseudocapacitive performance under deformation. These advances collectively highlight that the functionality of polymer–MXene composites arises from the interplay between interfacial chemistry, transport regulation, and structural preservation.

Moving beyond laboratory-scale demonstrations toward practical applications will require coordinated efforts across several fronts. Scalable synthesis routes that preserve MXene quality remain to be further developed, while polymer formulations should be optimized for both electrochemical performance and manufacturability. Standardized testing protocols that capture real operating conditions, including mechanical stress, temperature variations, and long-term cycling, are important for benchmarking device performance. Furthermore, the integration of operando characterization and data-driven modeling will accelerate the discovery of next-generation materials by enabling real-time insight into degradation mechanisms and predictive optimization of composite structures.

Ultimately, the convergence of advanced synthesis, interfacial engineering, and intelligent design strategies will position polymer–MXene composites as an important materials platform for next-generation energy storage systems. Their ability to simultaneously address conductivity, stability, and mechanical adaptability provides a pathway toward safe, efficient, and durable devices for applications ranging from grid-scale storage to wearable electronics.

## Figures and Tables

**Table 1:** Representative design strategies in polymer–MXene composites for Na-ion battery anodes, highlighting their roles in regulating structure, ion transport, and electrochemical stability.

Design Strategy	Representative System/Scope	Polymer Contribution	Main Effect on Na Storage	Suggested Use in Section 3	Ref.
Porosity engineering of Ti_3_C_2_T_x_	Porous or expanded-layer Ti_3_C_2_T_x_ MXene anodes	Provides structural support	Increases ion-accessible surface area and shortens Na^+^ diffusion pathways, improving rate capability	Section 3.1 Porosity engineering and interlayer preservation	[43]
Polymer-derived carbon decoration	Polymer-derived carbon-coated or heterostructured MXene-based anodes	Acts as carbon precursor and interfacial stabilizer	Suppresses restacking, enhances electronic conductivity, and buffers structural stress during cycling	Section 3.2 Polymer-derived carbon coatings and heterostructures	[10,11,44]
Polymer-assisted ion transport (solid/gel systems)	MXene electrodes integrated with polymer electrolytes or gel phases	Provides segmental mobility, salt dissociation, and interfacial contact	Improves Na^+^ transport, reduces interfacial resistance, and stabilizes ion-conduction pathways	Section 3.3 Solvent co-intercalation and segmental dynamics	[29,31,45]
Interlayer expansion and heterostructure design	Expanded-layer MXene heterostructures combined with binders or coatings	Supports interlayer spacing control and structural integrity	Enhances ion accessibility, maintains layered structure, and improves cycling stability	Bridge between Section 3.1 and Section 3.2	[10,11,40,43]
Atomic-scale engineering of MXene lattices	Vacancy-rich V_2_CT_x_ or Nb_2_CT_x_	Provides structural stabilization for defect-rich MXene sheets	Increases active sites for ion adsorption; facilitates faster Na+ diffusion via ordered vacancies	Section 3.1 and Section 3.2 (Lattice and Porosity Control)	[23,24,25,26]

**Table 2:** Representative interfacial engineering strategies in polymer- and MXene-enabled Zn anodes, highlighting their roles in regulating Zn^2+^ transport, interfacial stability, and plating/stripping reversibility.

Strategy	Representative System/Scope	Polymer Contribution	MXene or Inorganic Role	Main Interfacial Effect	Suggested Use in Section 4	Ref.
Polymer electrolyte membrane	Functional polymer membrane for Zn anode protection	Regulates local solvation, reduces water activity, and provides coordination sites for Zn^2+^	None is dominant in the baseline membrane concept	Suppresses HER and promotes uniform Zn nucleation	Section 4.1 Suppression of hydrogen evolution and dendrite growth	[12,16]
Polymer–MXene hydrogel interphase	Ti_3_C_2_T_x_–CNF ionotronic hydrogel	Provides mechanical confinement and ion-flux guidance through the polymer network	MXene contributes conductive pathways and interfacial homogenization	Suppresses dendritic growth and stabilizes Zn plating/stripping	Section 4.1 Suppression of hydrogen evolution and dendrite growth	[32,49]
Polymer–inorganic hybrid interphase	Zn silicate/polymer-based protective layer	Maintains conformal contact and accommodates interfacial strain	Inorganic phase acts as a rigid barrier and redistributes ion flux	Improves corrosion resistance and dendrite suppression	Section 4.2 Advanced polymer–inorganic hybrid interphases	[10,13]
MXene-enabled catalytic interphase	MXene-based interfacial regulation layer	Supports local coordination control when integrated with polymeric domains	MXene accelerates desolvation and modulates interfacial reactions	Improves Zn^2+^ kinetics and stabilizes deposition behavior	Section 4.3 Mechanistic insights into Zn solvation and transport	[18,48]
Mechanistic overview of MXene-based AZIB stabilization	Mechanistic review perspective on MXene-enabled Zn interfaces	Provides design context for polymer-assisted interphases	Establishes mechanistic interpretation of ion-flux regulation and interfacial catalysis	Connects solvation control, transport regulation, and durability	Bridge across Section 4.1, Section 4.2 and Section 4.3	[16,49]
Vacancy-enabled catalytic regulation	Ordered-vacancy V_2_CT_x_ MXene	Regulates local Zn^2+^ coordination and maintains interfacial contact	Accelerates Zn^2+^ desolvation through vacancy-rich catalytic sites	Reduces nucleation overpotential and promotes uniform Zn deposition	Section 4.1 and Section 4.3 (Dendrite Suppression and Kinetics)	[23]

**Table 3:** Comparison of shared and system-specific stabilization mechanisms in MXene/polymer heterostructures for Na-ion and Zn-ion battery systems.

Energy Storage System	Shared Mechanisms	System-Specific Mechanisms	Role of Polymer Component	Ref.
Na-ion batteries	Interfacial passivation, suppression of oxygen/water diffusion, structural preservation, and coupled ion/electron transport regulation	Interlayer expansion for Na^+^ transport, preservation of interlayer galleries, maintenance of ion-accessible channels, reduced diffusion resistance	Interlayer spacing control and structural stabilization through steric hindrance and mechanical separation of MXene nanosheets	[9,21,31,55,56]
Zn-ion batteries	Interfacial passivation, suppression of parasitic reactions, ion-flux homogenization, and stabilization of Zn plating/stripping behavior	Zn^2+^ desolvation regulation, modulation of local solvation structure, reduction of the desolvation kinetic barrier, uniform Zn nucleation and deposition, dendrite suppression, stabilization of Zn plating/stripping processes	Ion-coordination-assisted interfacial regulation and mechanical confinement for stable Zn deposition	[9,12,17,18,21,55,56]

## Data Availability

No new data were created or analyzed in this study. Data sharing is not applicable to this article as all data discussed are available from previously published sources cited within the manuscript.
